# Supplementary material for: iChip-Inspired Isolation, Bioactivities and Dereplication of Actinomycetota from Portuguese Beach Sediments
Source: Microorganisms. 2022 Jul 20;10(7):1471. doi: 10.3390/microorganisms10071471 (PMC9319460; doi:10.3390/microorganisms10071471)
Supplement: Supplementary file 1 [file microorganisms-10-01471-s001.zip › microorganisms-1821880-supplementary.pdf]

Table S1 - Isolated strains ID, affiliation, phyla and 16S rRNA gene GenBank accession number.

Strain ID	Affiliation (according to EzBioCloud)		Phylum	GenBank 16S rRNA gene Accession Number
	%	Taxonomic unit		
PMIC_1E12B	100.00	*Arthrobacter gandavensis* R 5812	*Actinomycetota*	MW740015
PMIC_1F10B.1	100.00	*Arthrobacter gandavensis* R 5812	*Actinomycetota*	MW740020
PMIC_1F10C.1	100.00	*Arthrobacter gandavensis* R 5812	*Actinomycetota*	MW740021
PMIC_2F9	100.00	*Arthrobacter gandavensis* R 5812	*Actinomycetota*	MW740074
PMIC_1D8B	100.00	*Corynebacterium marinum* DSM 44953	*Actinomycetota*	MW740010
PMIC_1C11A	100.00	*Dietzia maris* DSM 43672	*Actinomycetota*	MW740000
PMIC_1C5B.1	100.00	*Dietzia maris* DSM 43672	*Actinomycetota*	MW740004
PMIC_1H7A	100.00	*Kocuria polaris* CMS 76or	*Actinomycetota*	MW740027
PMIC_1C1B	98.48	*Microbacterium diaminobutyricum* RZ63	*Actinomycetota*	MW740003
PMIC_1A10A	100.00	*Microbacterium flavum* YM18-098	*Actinomycetota*	MW739985
PMIC_1C7A	99.92	*Microbacterium phyllosphaerae* DSM 13468	*Actinomycetota*	MW740005
PMIC_1E12A	99.44	*Micromonospora citrea* DSM 43903	*Actinomycetota*	MW740014
PMIC_1A10B	99.82	*Nocardia nova* NBRC 15556	*Actinomycetota*	MW739986
PMIC_1A11B.1	99.92	*Nocardiopsis alba* DSM 43377	*Actinomycetota*	MW739987
PMIC_1A11B.2	100.00	*Nocardiopsis alba* DSM 43377	*Actinomycetota*	MW739988
PMIC_1F6A.1	99.77	*Nocardiopsis alba* DSM 43377	*Actinomycetota*	MW740024
PMIC_1F6A.2	99.76	*Nocardiopsis alba* DSM 43377	*Actinomycetota*	MW740025
PMIC_1F6A3	99.76	*Nocardiopsis alba* DSM 43377	*Actinomycetota*	MW740026
PMIC_2A11A.1	99.92	*Nocardiopsis alba* DSM 43377	*Actinomycetota*	MW740030
PMIC_2A11A.2	100.00	*Nocardiopsis alba* DSM 43377	*Actinomycetota*	MW740031
PMIC_2A11B.1	100.00	*Nocardiopsis alba* DSM 43377	*Actinomycetota*	MW740032
PMIC_2A11B.2	100.00	*Nocardiopsis alba* DSM 43377	*Actinomycetota*	MW740033
PMIC_2A11B.3	100.00	*Nocardiopsis alba* DSM 43377	*Actinomycetota*	MW740034
PMIC_2F6A	99.76	*Nocardiopsis alba* DSM 43377	*Actinomycetota*	MW740071
PMIC_2F6B	99.76	*Nocardiopsis alba* DSM 43377	*Actinomycetota*	MW740072
PMIC_2F6C	99.77	*Nocardiopsis alba* DSM 43377	*Actinomycetota*	MW740073
PMIC_2H2A	100.00	*Nocardiopsis alba* DSM 43377	*Actinomycetota*	MW740077
PMIC_2H2C.1	100.00	*Nocardiopsis alba* DSM 43377	*Actinomycetota*	MW740078
PMIC_2H2C.2	100.00	*Nocardiopsis alba* DSM 43377	*Actinomycetota*	MW740079
PMIC_1D11B	99.84	*Nocardiopsis prasina* DSM 43845	*Actinomycetota*	MW740009
PMIC_2B1A	99.85	*Nocardiopsis prasina* DSM 43845	*Actinomycetota*	MW740039
PMIC_2B1C	99.82	*Nocardiopsis prasina* DSM 43845	*Actinomycetota*	MW740040
PMIC_2B1D	99.85	*Nocardiopsis prasina* DSM 43845	*Actinomycetota*	MW740041
PMIC_2D10A	99.84	*Nocardiopsis prasina* DSM 43845	*Actinomycetota*	MW740052
PMIC_2D10B.1	99.75	*Nocardiopsis prasina* DSM 43845	*Actinomycetota*	MW740053
PMIC_2D10B.2	99.84	*Nocardiopsis prasina* DSM 43845	*Actinomycetota*	MW740054
PMIC_2D10C	99.84	*Nocardiopsis prasina* DSM 43845	*Actinomycetota*	MW740055
PMIC_2F12A	99.82	*Nocardiopsis prasina* DSM 43845	*Actinomycetota*	MW740068
PMIC_2C3B.1	99.11	*Nocardiopsis umidischolae* 66/93	*Actinomycetota*	MW740045

PMIC_2C3B.2	99.11	*Nocardiopsis umidischolae* 66/93	*Actinomycetota*	MW740046
PMIC_2C3B.3	99.19	*Nocardiopsis umidischolae* 66/93	*Actinomycetota*	MW740047
PMIC_2C3B.4	99.20	*Nocardiopsis umidischolae* 66/93	*Actinomycetota*	MW740048
PMIC_2H6	99.81	*Plantibacter flavus* VKM Ac-2504	*Actinomycetota*	MW740080
PMIC_1E10C	99.69	*Rhodococcus coprophilus* NBRC 100603	*Actinomycetota*	MW740013
PMIC_2E10	99.69	*Rhodococcus coprophilus* NBRC 100603	*Actinomycetota*	MW740062
PMIC_1E9B	100.00	*Rhodococcus erythropolis* NBRC 15567	*Actinomycetota*	MW740017
PMIC_2E9B.1	100.00	*Rhodococcus erythropolis* NBRC 15567	*Actinomycetota*	MW740066
PMIC_2E9C	99.76	*Rhodococcus qingshengii* JCM 15477	*Actinomycetota*	MW740067
PMIC_2A12A.1	99.68	*Streptomyces albidoflavus* DSM 40455	*Actinomycetota*	MW740035
PMIC_2A12A.2	99.69	*Streptomyces albidoflavus* DSM 40455	*Actinomycetota*	MW740036
PMIC_2A12B.1	99.77	*Streptomyces albidoflavus* DSM 40455	*Actinomycetota*	MW740037
PMIC_2A12B.2	99.76	*Streptomyces albidoflavus* DSM 40455	*Actinomycetota*	MW740038
PMIC_2C12	99.69	*Streptomyces albidoflavus* DSM 40455	*Actinomycetota*	MW740042
PMIC_1A3B.1	100.00	*Streptomyces albogriseolus* NRRL B-1305	*Actinomycetota*	MW739992
PMIC_1A3B.2	100.00	*Streptomyces albogriseolus* NRRL B-1305	*Actinomycetota*	MW739993
PMIC_1A3C	100.00	*Streptomyces albogriseolus* NRRL B-1305	*Actinomycetota*	MW739994
PMIC_1A8C	99.66	*Streptomyces albogriseolus* NRRL B-1305	*Actinomycetota*	MW739996
PMIC_1C12A	99.67	*Streptomyces albogriseolus* NRRL B-1305	*Actinomycetota*	MW740001
PMIC_1C12B	99.68	*Streptomyces albogriseolus* NRRL B-1305	*Actinomycetota*	MW740002
PMIC_2G2A	99.68	*Streptomyces ambofaciens* ATCC 23877	*Actinomycetota*	MW740075
PMIC_2G8C	99.68	*Streptomyces ambofaciens* ATCC 23877	*Actinomycetota*	MW740076
PMIC_1C8A	99.69	*Streptomyces ardesiacus* NRRL B-1773	*Actinomycetota*	MW740006
PMIC_2C8A	99.69	*Streptomyces ardesiacus* NRRL B-1773	*Actinomycetota*	MW740049
PMIC_2C8B	99.70	*Streptomyces ardesiacus* NRRL B-1773	*Actinomycetota*	MW740050
PMIC_2D8A	100.00	*Streptomyces ardesiacus* NRRL B-1773	*Actinomycetota*	MW740060
PMIC_2D8B	100.00	*Streptomyces ardesiacus* NRRL B-1773	*Actinomycetota*	MW740061
PMIC_1A8B	99.43	*Streptomyces flavoviridis* NBRC 12772	*Actinomycetota*	MW739995
PMIC_1D9A	99.80	*Streptomyces griseoflavus* LMG 19344	*Actinomycetota*	MW740011
PMIC_1D9B	99.75	*Streptomyces griseoflavus* LMG 19344	*Actinomycetota*	MW740012
PMIC_1D11A.1	99.85	*Streptomyces hydrogenans* NBRC 13475	*Actinomycetota*	MW740007
PMIC_1D11A.2	99.84	*Streptomyces hydrogenans* NBRC 13475	*Actinomycetota*	MW740008
PMIC_1I1A	100.00	*Streptomyces hydrogenans* NBRC 13475	*Actinomycetota*	MW740028
PMIC_1I1B	100.00	*Streptomyces hydrogenans* NBRC 13475	*Actinomycetota*	MW740029
PMIC_2C8C	99.57	*Streptomyces hydrogenans* NBRC 13475	*Actinomycetota*	MW740051
PMIC_2D11A.1	99.77	*Streptomyces hydrogenans* NBRC 13475	*Actinomycetota*	MW740056
PMIC_2D11A.2	99.69	*Streptomyces hydrogenans* NBRC 13475	*Actinomycetota*	MW740057
PMIC_2D11B	99.84	*Streptomyces hydrogenans* NBRC 13475	*Actinomycetota*	MW740058
PMIC_2D11C	99.70	*Streptomyces hydrogenans* NBRC 13475	*Actinomycetota*	MW740059
PMIC_1F12A	100.00	*Streptomyces setonii* NRRL ISP-5322	*Actinomycetota*	MW740022
PMIC_1F12B	100.00	*Streptomyces setonii* NRRL ISP-5322	*Actinomycetota*	MW740023
PMIC_2F12B	100.00	*Streptomyces setonii* NRRL ISP-5322	*Actinomycetota*	MW740069
PMIC_2F12C	100.00	*Streptomyces setonii* NRRL ISP-5322	*Actinomycetota*	MW740070
PMIC_1A3A.1	99.85	*Streptomyces xiamenensis* MCCC 1A01550	*Actinomycetota*	MW739989
PMIC_1A3A.2	99.82	*Streptomyces xiamenensis* MCCC 1A01550	*Actinomycetota*	MW739990
PMIC_1A3A.3	99.82	*Streptomyces xiamenensis* MCCC 1A01550	*Actinomycetota*	MW739991
PMIC_1B3A.1	99.52	*Streptomyces xiamenensis* MCCC 1A01550	*Actinomycetota*	MW739997
PMIC_1B3A.2	99.54	*Streptomyces xiamenensis* MCCC 1A01550	*Actinomycetota*	MW739998

PMIC_1B3A.3	99.55	*Streptomyces xiamenensis* MCCC 1A01550	*Actinomycetota*	MW739999
PMIC_2C2A	99.84	*Streptomyces xiamenensis* MCCC 1A01550	*Actinomycetota*	MW740043
PMIC_2C2B	99.81	*Streptomyces xiamenensis* MCCC 1A01550	*Actinomycetota*	MW740044
PMIC_2E9A.1	99.84	*Streptomyces xiamenensis* MCCC 1A01550	*Actinomycetota*	MW740063
PMIC_2E9A.2	99.85	*Streptomyces xiamenensis* MCCC 1A01550	*Actinomycetota*	MW740064
PMIC_2E9A.3	99.85	*Streptomyces xiamenensis* MCCC 1A01550	*Actinomycetota*	MW740065
PMIC_1E9A	99.82	*Streptomyces xiamenensis* MCCC1A01550	*Actinomycetota*	MW740016
PMIC_1E9C	99.84	*Streptomyces xiamenensis* MCCC1A01550	*Actinomycetota*	MW740018
PMIC_1E9D	99.81	*Streptomyces xiamenensis* MCCC1A01550	*Actinomycetota*	MW740019
PMIC_1E1A.1	99.52	*Aquimarina algiphila* 9Alg 151	*Bacteroidota*	MW740084
PMIC_1E1A.2	99.91	*Aquimarina amphilecti* 92V	*Bacteroidota*	MW740085
PMIC_1A1	99.06	*Aquimarina muelleri* KMM 6020	*Bacteroidota*	MW740081
PMIC_1E11B.2	99.59	*Arenibacter aquaticus* GUO666	*Bacteroidota*	MW740083
PMIC_2G2B	99.74	*Arenibacter aquaticus* GUO666	*Bacteroidota*	MW740087
PMIC_1B2	99.92	*Catalinimonas alkaloidigena* CNU-914	*Bacteroidota*	MW740082
PMIC_2E5A	99.67	*Tenacibaculum gallaicum* A37.1	*Bacteroidota*	MW740086
PMIC_2C11	100.00	*Alkalihalobacillus algicola* KMM 3737	*Bacillus*	MW740107
PMIC_2G8A.1	100.00	*Alkalihalobacillus hwajinpoensis* SW-72	*Bacillus*	MW740108
PMIC_1B9B.1	100.00	*Bacillus aryabhattai* B8W22	*Bacillus*	MW740089
PMIC_1B9B.2	100.00	*Bacillus aryabhattai* B8W22	*Bacillus*	MW740090
PMIC_1E11A.1	100.00	*Bacillus aryabhattai* B8W22	*Bacillus*	MW740093
PMIC_1E11B.1	100.00	*Bacillus aryabhattai* B8W22	*Bacillus*	MW740094
PMIC_1E1B.1	100.00	*Bacillus aryabhattai* B8W22	*Bacillus*	MW740095
PMIC_1E1B.2	100.00	*Bacillus aryabhattai* B8W22	*Bacillus*	MW740096
PMIC_2A10B.3	100.00	*Bacillus aryabhattai* B8W22	*Bacillus*	MW740102
PMIC_2B9A.1	100.00	*Bacillus horikoshii* DSM 8719	*Bacillus*	MW740104
PMIC_2B9A.2	100.00	*Bacillus horikoshii* DSM 8719	*Bacillus*	MW740105
PMIC_2B9A.3	100.00	*Bacillus horikoshii* DSM 8719	*Bacillus*	MW740106
PMIC_1E10A	99.71	*Bacillus licheniformis* ATCC 14580	*Bacillus*	MW740092
PMIC_1F10B.2	99.93	*Bacillus megaterium* NBRC 15308	*Bacillus*	MW740097
PMIC_1F10B.3	99.92	*Bacillus megaterium* NBRC 15308	*Bacillus*	MW740098
PMIC_1F10C.2	99.92	*Bacillus megaterium* NBRC 15308	*Bacillus*	MW740099
PMIC_2A10B.1	100.00	*Bacillus megaterium* NBRC 15308	*Bacillus*	MW740101
PMIC_1A11C	100.00	*Bacillus mycoides* DSM 2048	*Bacillus*	MW740088
PMIC_2A11C	100.00	*Bacillus mycoides* DSM 2048	*Bacillus*	MW740103
PMIC_2H10A	99.92	*Bacillus pumilus* ATCC 7061	*Bacillus*	MW740109
PMIC_1D12	100.00	*Bacillus toyonensis* BCT-7112	*Bacillus*	MW740091
PMIC_2A10A	99.85	*Fictibacillus phosphorivorans* Ca7	*Bacillus*	MW740100
PMIC_2H3	100.00	*Cobetia amphilecti* KMM 1561	*Pseudomonadota*	MW740140
PMIC_1D2B.1	99.85	*Cobetia marina* JCM 21022	*Pseudomonadota*	MW740118
PMIC_1D2B.3	100.00	*Cobetia marina* JCM 21022	*Pseudomonadota*	MW740120
PMIC_2F12E	99.23	*Henriciella algicola* CCUG 67844	*Pseudomonadota*	MW740134
PMIC_1E8A	99.33	*Limimaricola cinnabarinus* LL-001	*Pseudomonadota*	MW740123
PMIC_1E8C	99.33	*Limimaricola cinnabarinus* LL-001	*Pseudomonadota*	MW740124
PMIC_1H8A	99.30	*Limimaricola soesokkakensis* CECT 8367	*Pseudomonadota*	MW740126
PMIC_1C11B	100.00	*Marinobacter litoralis* SW-45	*Pseudomonadota*	MW740115
PMIC_1E1A.3	100.00	*Marinobacter litoralis* SW-45	*Pseudomonadota*	MW740122
PMIC_1A8A	100.00	*Phaeobacter porticola* P97	*Pseudomonadota*	MW740110

PMIC_2H5C	99.92	*Providencia vermicola* OP1	*Pseudomonadota*	MW740142
PMIC_1D2B.2	100.00	*Pseudoalteromonas atlantica* NBRC 103033	*Pseudomonadota*	MW740119
PMIC_1B3B	100.00	*Pseudoalteromonas carrageenovora* IAM 12662	*Pseudomonadota*	MW740111
PMIC_1C10	100.00	*Pseudoalteromonas carrageenovora* IAM12662	*Pseudomonadota*	MW740114
PMIC_2C3A	100.00	*Pseudoalteromonas carrageenovora* IAM12662	*Pseudomonadota*	MW740127
PMIC_2H5B	99.84	*Pseudoalteromonas carrageenovora* IAM12662	*Pseudomonadota*	MW740141
PMIC_1F6B	100.00	*Pseudoalteromonas neustonica* PAMC28425	*Pseudomonadota*	MW740125
PMIC_2C8D	99.13	*Pseudoalteromonas prydzensis* MB8-11	*Pseudomonadota*	MW740129
PMIC_1B5B.1	100.00	*Pseudoalteromonas tetraodonis* GFC	*Pseudomonadota*	MW740112
PMIC_1B5B.2	100.00	*Pseudoalteromonas tetraodonis* GFC	*Pseudomonadota*	MW740113
PMIC_1C5B.2	99.35	*Psychrobacter cryohalolentis* K5	*Pseudomonadota*	MW740116
PMIC_2G8A.2	99.52	*Psychrobacter cryohalolentis* K5	*Pseudomonadota*	MW740138
PMIC_2G8B	99.53	*Psychrobacter cryohalolentis* K5	*Pseudomonadota*	MW740139
PMIC_1D8D.1	100.00	*Psychrobacter nivimaris* 88/2-7	*Pseudomonadota*	MW740121
PMIC_2D8E	100.00	*Psychrobacter nivimaris* 88/2-7	*Pseudomonadota*	MW740131
PMIC_2E9B.2	100.00	*Psychrobacter nivimaris* 88/2-7	*Pseudomonadota*	MW740133
PMIC_1D1B.1	100.00	*Sulfitobacter pontiacus* DSM 10014	*Pseudomonadota*	MW740117
PMIC_2D1A	100.00	*Sulfitobacter pontiacus* DSM 10014	*Pseudomonadota*	MW740130
PMIC_2E5B	100.00	*Sulfitobacter pontiacus* DSM 10014	*Pseudomonadota*	MW740132
PMIC_2G1A.1	100.00	*Tritonibacter mobilis* subsp. *pelagius* NBRC 102038	*Pseudomonadota*	MW740135
PMIC_2G1A.2	100.00	*Tritonibacter mobilis* subsp. *pelagius* NBRC 102038	*Pseudomonadota*	MW740136
PMIC_2G1B	100.00	*Tritonibacter mobilis* subsp. *pelagius* NBRC 102038	*Pseudomonadota*	MW740137
PMIC_2C5A	99.93	*Vibrio toranzoniae* Vb 10.8	*Pseudomonadota*	MW740128

Table S2 - Total and per medium distribution of isolated strains by genera.

Phylum	Genus	Total Isolates	M600 Isolates	MA Isolates
Actinomycetota	*Arthrobacter*	4	3	1
	Corynebacterium	1	1	0
	Dietzia	2	2	0
	Kocuria	1	1	0
	Microbacterium	3	3	0
	Micromonospora	1	1	0
	Nocardia	1	1	0
	Nocardiopsis	29	6	23
	Rhodococcus	5	2	3
	Streptomyces	48	25	23
	Plantibacter	1	0	1
Bacteroidota	*Aquimarina*	3	3	0
	Arenibacter	2	1	1
	Catalinimonas	1	1	0
	Tenacibaculum	1	0	1
Bacillota	*Alkalihalobacillus*	2	0	2
	Bacillus	19	12	7
	Fictibacillus	1	0	1
Pseudomonadota	*Cobetia*	3	2	1
	Henriciella	1	0	1
	Limimaricola	3	3	0
	Marinobacter	2	2	0
	Phaeobacter	1	1	0
	Providencia	1	0	1
	Pseudoalteromonas	9	6	3
	Psychrobacter	6	2	4
	Sulfitobacter	3	1	2
	Tritonibacter	3	0	3
	Vibrio	1	0	1

Table S3 - All compounds putatively identified and non-identified molecules present in the extracts.

Strain ID	Taxonomic unit	Medium	Putatively Detected Molecules	Non-Identified Molecules
PMIC_1E12B	*Arthrobacter gandavensis*	M607	cyclo(Pro-Tyr), cyclo(L-Leu-L-Pro), cyclo(Phe-Pro), 1H-indole-3-acetic acid, cyclo(Phe-Lxx), lauric diethanolamide	–
		OSMAC 1:10 M607	–	–
		OSMAC M607	–	–
		OSMAC M600	–	–
		OSMAC MA	–	–
		OSMAC CGY	–	–
PMIC_1A10B	*Nocardia nova*	M607	lauric diethanolamide, N-acetyltyramine, cyclo(L-Leu-L-Pro), cyclo(Phe-Pro), 1H-indole-3-acetic acid	–
		OSMAC 1:10 M607	taurocholic acid, linoleic acid, N-N-dimethyladenosine, N-acetyltyramine, cyclo(L-Leu-L-Pro), 1H-indole-3-acetic acid, cyclo(Phe-Pro), cyclo(Pro-Trp)	$C_9H_{10}N_2O$; $C_{15}H_{24}O_3$
		OSMAC M607	adenosine, cyclo(Pro-Tyr), cyclo(L-Leu-L-Pro), 1H-indole-3-acetic acid, cyclo(Phe-Pro), cyclo(Pro-Trp), taurocholic acid, lauric diethanolamide	$C_{27}H_{54}N_{10}O_{10}$
		OSMAC M600	adenosine, cyclo(Pro-Tyr), N-acetyltyramine, cyclo(L-Leu-L-Pro), cyclo(Pro-Trp), cyclo(Phe-Pro), cyclo(Phe-Val), cyclo(Phe-Lxx), taurocholic acid, lauramidopropyl betaine, lauric diethanolamide	$C_{22}H_{44}O_{12}$
		OSMAC MA	adenosine, cyclo(Pro-Tyr), N-acetyltyramine, cyclo(L-Leu-L-Pro), cyclo(Phe-Pro), N-[2-(1H-Indol-3-yl)-2-oxoethyl]acetamide, cyclo(Pro-Trp), cyclo(Phe-Val), N-(2-phenylethyl)acetamide, cyclo(Phe-Lxx), taurocholic acid, lauric diethanolamide	–

		OSMAC CGY	N-N-dimethyladenosine, N-acetyltyramine, cyclo(L-Leu-L-Pro), 1H-indole-3-acetic acid, cyclo(Phe-Pro), taurocholic acid, cyclo(Phe-Lxx)	$C_{22}H_{44}O_{12}$; $C_{24}H_{48}O_{13}$
PMIC_1A11B.2	*Nocardiopsis alba*	M607	N-acetyltyramine, cyclo(Pro-Tyr), cyclo(L-Leu-L-Pro), 1H-indole-3-acetic acid, cyclo(Phe-Lxx), germicidin A, lauric diethanolamide	$C_{12}H_{25}NO_3$, $C_{14}H_{29}NO_3$
		OSMAC 1:10 M607	–	–
		OSMAC M607	–	–
		OSMAC M600	–	–
		OSMAC MA	–	–
		OSMAC CGY	–	–
PMIC_2A11B.1	*Nocardiopsis alba*	M607	cyclo(Pro-Trp), N-acetyltyramine, cyclo(L-Leu-L-Pro), 1H-indole-3-acetic acid, lumichrome, lauric diethanolamide, germicidin A	–
		OSMAC 1:10 M607	–	–
		OSMAC M607	–	–
		OSMAC M600	N-acetyltyramine, cyclo(L-Leu-L-Pro), 1H-indole-3-acetic acid, cyclo(Pro-Trp), germicidin A, taurocholic acid	–
		OSMAC MA	–	–
		OSMAC CGY	N-acetyltyramine, cyclo(L-Leu-L-Pro), cyclo(Phe-Pro), cyclo(Pro-Trp), taurocholic acid, germicidin A, antibiotic X 14952B	–
PMIC_1F6A.3	*Nocardiopsis alba*	M607	cyclo(Pro-Tyr), MDN-0100, cyclo(L-Leu-L-Pro), 1H-indole-3-acetic acid, cyclo(Phe-Pro), lauric diethanolamide	$C_{20}H_{31}NO_4S$
		OSMAC 1:10 M607	–	–
		OSMAC M607	–	–

		OSMAC M600	–	–
		OSMAC MA	–	–
		OSMAC CGY	–	–
PMIC_2H2C.2	*Nocardiopsis alba*	M607	cyclo(L-Leu-L-Pro), 1H-indole-3-acetic acid, cyclo(Phe-Pro), lauric diethanolamide	–
		OSMAC 1:10 M607	cyclo(L-Leu-L-Pro), taurocholic acid	$C_{27}H_{52}N_{10}O_{10}$
		OSMAC M607	–	–
		OSMAC M600	N-acetyltyramine, cyclo(L-Leu-L-Pro), cyclo(Phe-Pro), 1H-indole-3-acetic acid, cyclo(Pro-Trp), taurocholic acid, antibiotic X 14952B	$C_{15}H_{24}O_4$
		OSMAC MA	–	–
		OSMAC CGY	cyclo(Pro-Tyr), N-acetyltyramine, cyclo(L-Leu-L-Pro), cyclo(Phe-Pro), cyclo(Pro-Trp), N-(2-phenylethyl)acetamide, cyclo(Phe-Lxx), taurocholic acid, antibiotic X 14952B	$C_{15}H_{24}O_4$, $C_{16}H_{24}O_3$
PMIC_1E10C	*Rhodococcus coprophilus*	M607	cyclo(L-Leu-L-Pro), 1H-indole-3-acetic acid, cyclo(Phe-Pro), cyclo(Pro-Trp), lauric diethanolamide	$C_{14}H_{29}NO_3$
		OSMAC 1:10 M607	–	–
		OSMAC M607	–	–
		OSMAC M600	–	–
		OSMAC MA	–	–
		OSMAC CGY	–	–

PMIC_2C12	*Streptomyces albidoflavus*	M607	cyclo(Pro-Tyr), N-acetyltyramine, cyclo(L-Leu-L-Pro), 1H-indole-3-acetic acid, cyclo(Phe-Pro), germicidin G, lauric diethanolamide, MDN-0142 - surugamide A, ansalactam A	–
		OSMAC 1:10 M607	–	–
		OSMAC M607	–	–
		OSMAC M600	–	–
		OSMAC MA	–	–
		OSMAC CGY	–	–
PMIC_1A8B	*Streptomyces flavoviridis*	M607	N-acetyltyramine, cyclo(L-Leu-L-Pro), 1H-indole-3-acetic acid, cyclo(Phe-Pro), cyclo(Pro-Trp), N-(2-phenylethyl)acetamide, cyclo(Phe-Lxx), lauric diethanolamide	$C_{20}H_{13}N_3O_6$, $C_{23}H_{13}ClO_4S_2$
		OSMAC 1:10 M607	cyclo(L-Leu-L-Pro), taurocholic acid	$C_{23}H_{13}ClO_4S_2$
		OSMAC M607	cyclo(Pro-Tyr), N-acetyltyramine, cyclo(L-Leu-L-Pro), cyclo(Phe-Pro), taurocholic acid, lauric diethanolamide	–
		OSMAC M600	N-acetyltyramine, cyclo(L-Leu-L-Pro), cyclo(Phe-Pro), cyclo(Pro-Trp), cyclo(Phe-Val), cyclo(Phe-Lxx), taurocholic acid	$C_{20}H_{13}N_3O_6$, $C_{23}H_{13}ClO_4S_2$
		OSMAC MA	–	–
		OSMAC CGY	–	–
PMIC_1D9B	*Streptomyces griseoflavus*	M607	N-acetyltyramine, cyclo(L-Leu-L-Pro), 1H-indole-3-acetic acid, cyclo(Pro-Trp), N-(2-phenylethyl)acetamide, lauric diethanolamide, ansalactam A	–
		OSMAC 1:10 M607	N-acetyltyramine, taurocholic acid, lauramidopropyl betaine, lauric diethanolamide, ansalactam A	–

		OSMAC M607	cyclo(Pro-Tyr), N-acetyltyramine, cyclo(L-Leu-L-Pro), 1H-indole-3-acetic acid, cyclo(Phe-Pro), 3-acetylamino-N-2-thienylpropanamide, taurocholic acid, lauric diethanolamide, ansalactam A	–
		OSMAC M600	cyclo(Pro-Tyr), N-acetyltyramine, cyclo(L-Leu-L-Pro), 1H-indole-3-acetic acid, cyclo(Pro-Trp), 3-acetylamino-N-2-thienylpropanamide, taurocholic acid, lauramidopropyl betaine, lauric diethanolamide, ansalactam A	–
		OSMAC MA	–	–
		OSMAC CGY	N-acetyltyramine, cyclo(Phe-Pro), 3-acetylamino-N-2-thienylpropanamide, cyclo(Phe-Val), cyclo(Phe-Lxx), taurocholic acid, ansalactam A	–
PMIC_1I1A	*Streptomyces hydrogenans*	M607	N-N-dimethyladenosine, cyclo(Pro-Tyr), N-acetyltyramine, cyclo(L-Leu-L-Pro), 1H-indole-3-acetic acid, cyclo(Phe-Pro), cyclo(Pro-Trp), antibiotic MKN 003B, germicidin G, lauric diethanolamide, surugamide A	$C_{13}H_{22}O_3$
		OSMAC 1:10 M607	N-N-dimethyladenosine, cyclo(L-Leu-L-Pro), taurocholic acid, antibiotic MKN 003B, germicidin G, surugamide E, surugamide A	$C_{13}H_{22}O_3$
		OSMAC M607	–	–
		OSMAC M600	–	–
		OSMAC MA	–	–
		OSMAC CGY	N-N-dimethyladenosine, cyclo(Pro-Tyr), N-acetyltyramine, cyclo(L-Leu-L-Pro), cyclo(Phe-Pro), N-[2-(1H-Indol-3-yl)-2-oxoethyl]acetamide, cyclo(Pro-Trp), cyclo(Lxx-Lxx), cyclo(Phe-Lxx), taurocholic acid, antibiotic MKN 003B, germicidin G, lauramidopropyl betaine, surugamide E, surugamide A, blastmycin, antimycin A13, antimycin A11- SPA 8893A	$C_{13}H_{22}O_3$
PMIC_1F12B	*Streptomyces setonii*	M607	corynecin I, N-acetyltyramine, cyclo(Phe-Pro), 1H-indole-3-acetic acid, chloramphenicol, lauric diethanolamide	–

OSMAC 1:10 M607	N-acetyltyramine, cyclo(L-Leu-L-Pro), taurocholic acid, 3-(Hydroxymethyl)-2-(1-hydroxy-6-methylheptyl)butanolide	$C_{27}H_{53}N_5O_{10}$
OSMAC M607	–	–
OSMAC M600	N-N-dimethyladenosine, cyclo(L-Leu-L-Pro), cyclo(Phe-Pro), N-acetyltyramine, 1H-indole-3-acetic acid, taurocholic acid	–
OSMAC MA	–	–
OSMAC CGY	N-N-dimethyladenosine, N-acetyltyramine, cyclo(L-Leu-L-Pro), 1H-indole-3-acetic acid, N-[2-(1H-Indol-3-yl)-2-oxoethyl]acetamide, cyclo(Phe-Pro), cyclo(Phe-Lxx), taurocholic acid	$C_{22}H_{44}O_{12}$

Supplementary Figures

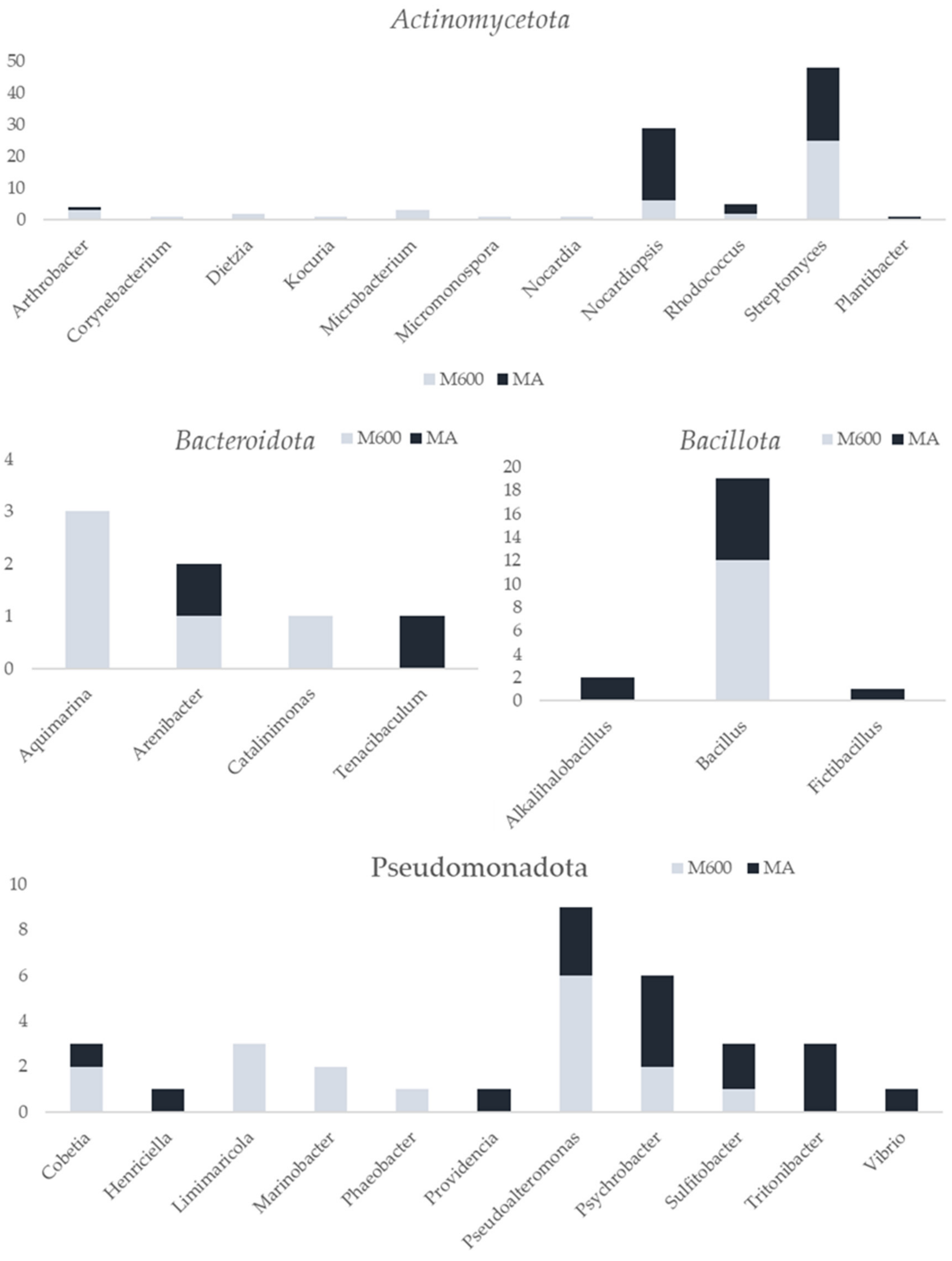

Figure S1 - Distribution of the genera of the obtained strains per medium used.

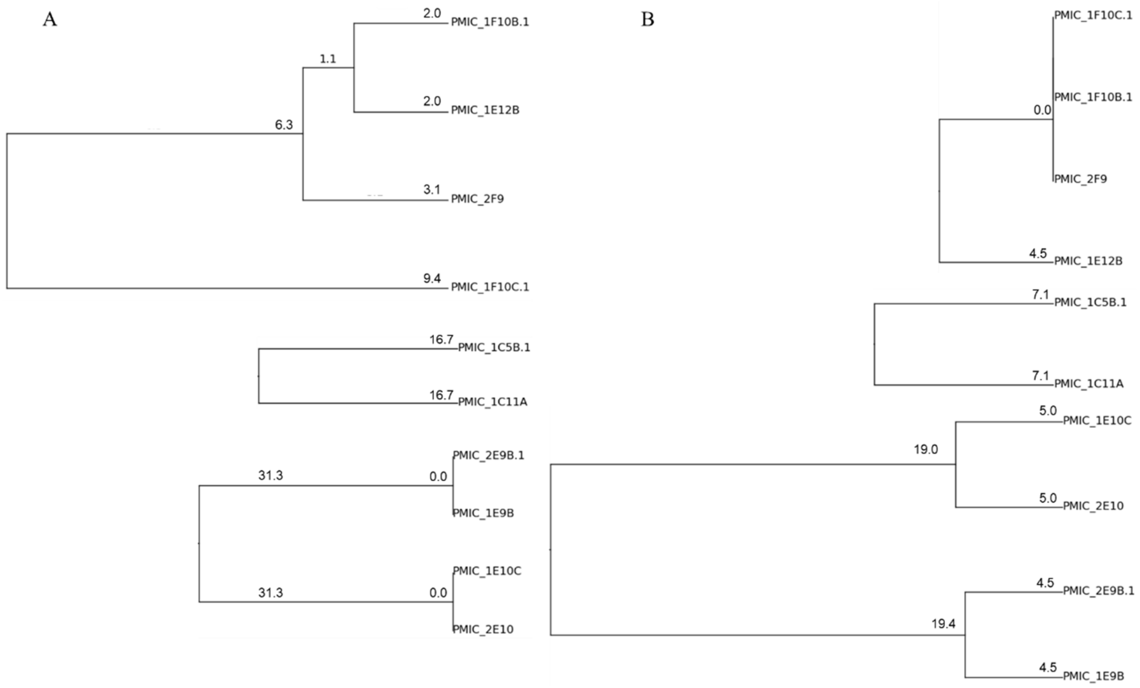

Figure S2 – (A) ERIC-PCR fingerprinting performed on strains belonging to *Arthorbacter gandavensis*, *Dietzia maris*, *Rhodococcus coprophilus* and *Rhodococcus erythropolis*. (B) BOX-PCR fingerprinting performed on strains belonging to *A. gandavensis*, *D. maris*, *R. coprophilus* and *R. erythropolis*.

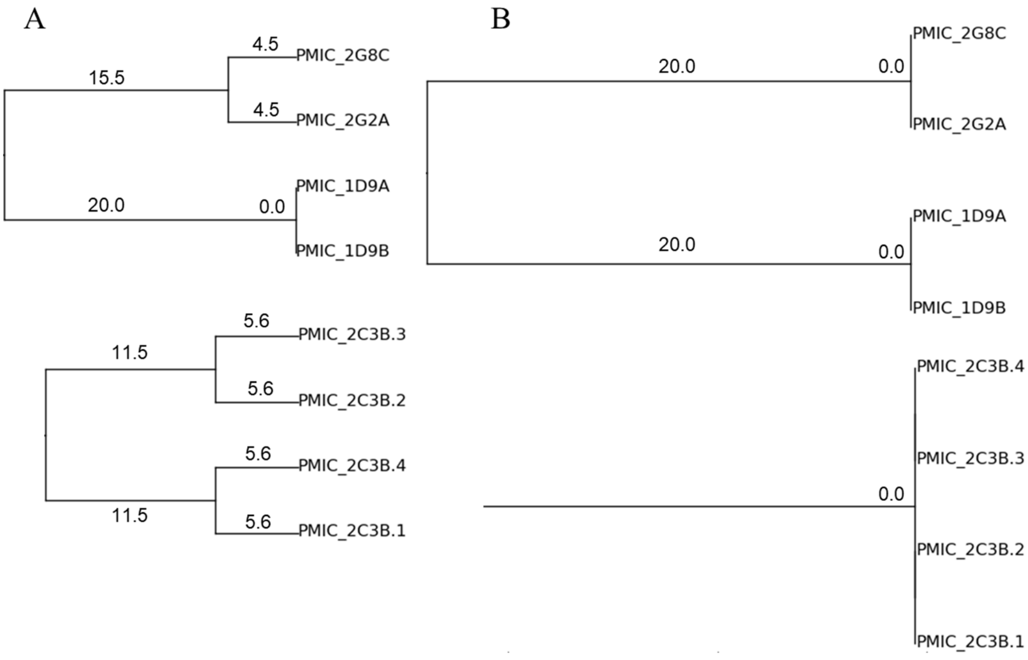

Figure S3 - (A) ERIC-PCR fingerprinting performed on strains belonging to *Streptomyces griseoflavus*, *Streptomyces ambofaciens* and *Nocardiopsis umidischolae*. (B) BOX-PCR fingerprinting performed on strains belonging to *S. griseoflavus*, *S. ambofaciens* and *N. umidischolae*.

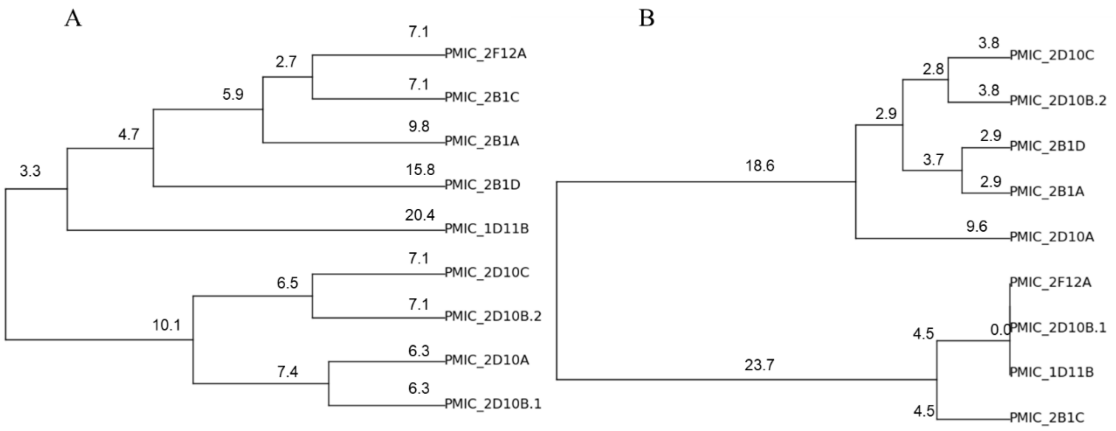

Figure S4 - (A) ERIC -PCR fingerprinting performed on strains belonging to *Nocardiopsis prasina*. (B) BOX-PCR fingerprinting performed on strains belonging to *N. prasina*.

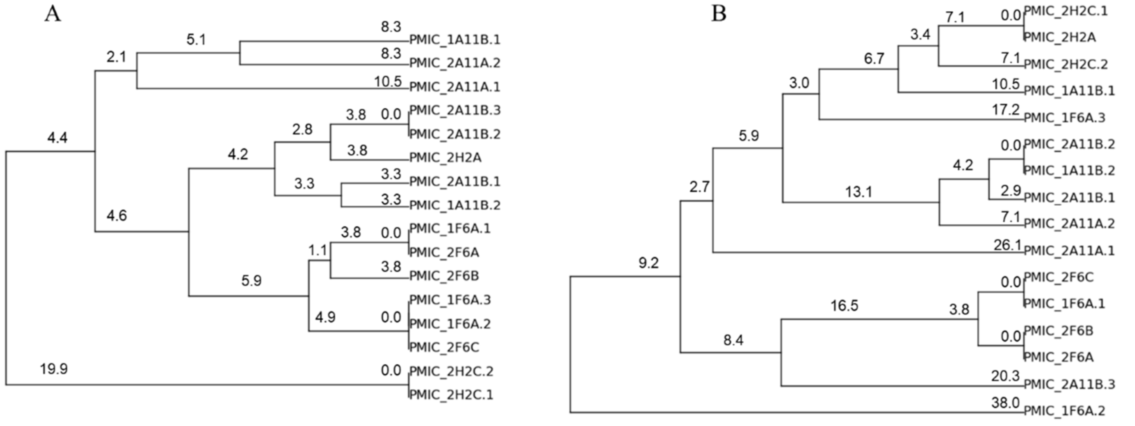

Figure S5 - (A) ERIC-PCR fingerprinting performed on strains belonging to *Nocardiopsis alba.* (B) BOX-PCR fingerprinting performed on strains belonging to *N. alba.*

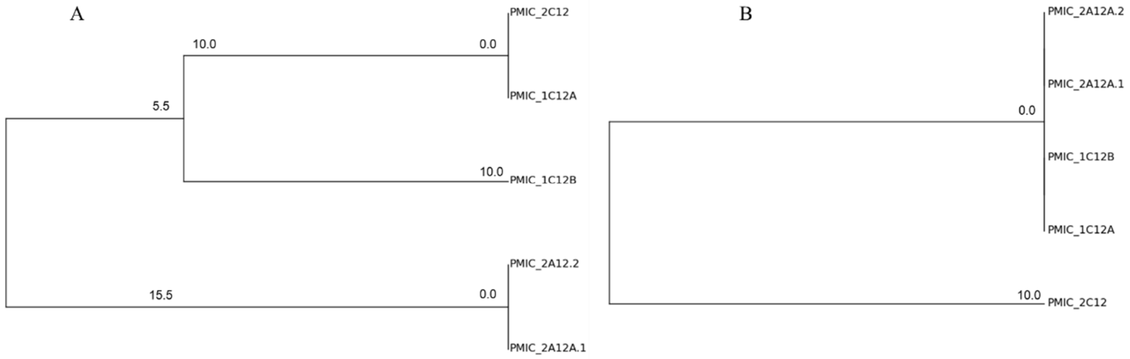

Figure S6 - (A) ERIC-PCR fingerprinting performed on strains belonging to *Streptomyces albidoflavus.* (B) BOX-PCR fingerprinting performed on strains belonging to *S. albidoflavus.*

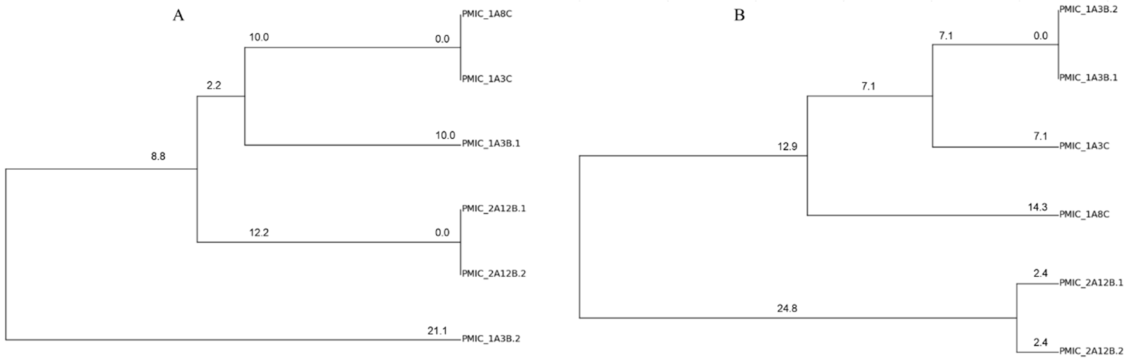

Figure S7 - (A) ERIC-PCR fingerprinting performed on strains belonging to *Streptomyces albogriseolus.* (B) BOX-PCR fingerprinting performed on strains belonging to *S. albogriseolus.*

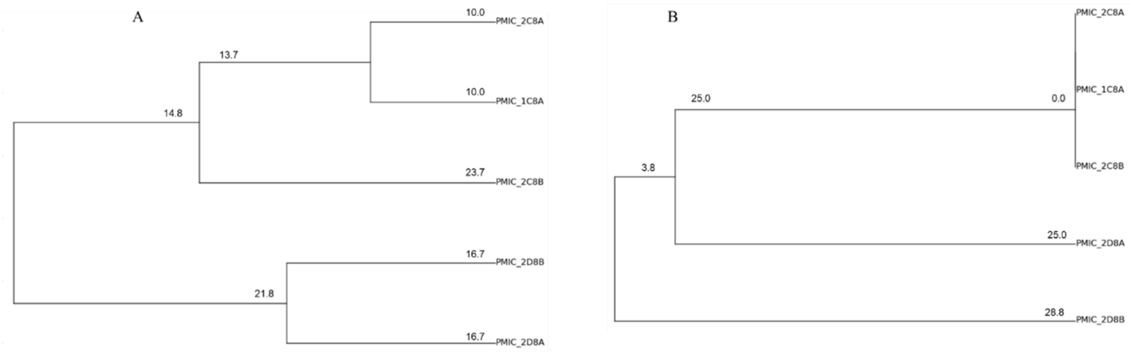

Figure S8 - (A) ERIC-PCR fingerprinting performed on strains belonging to *Streptomyces ardesiacus.* (B) BOX-PCR fingerprinting performed on strains belonging to *S. ardesiacus.*

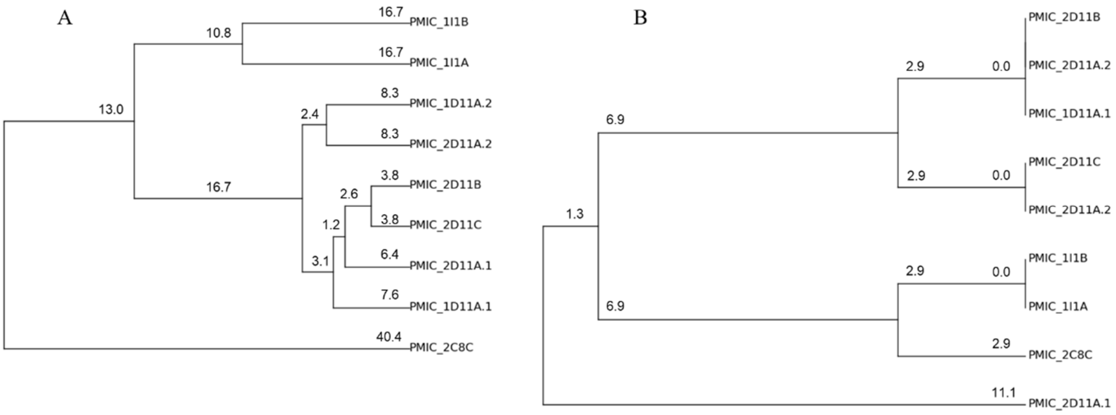

Figure S9 - (A) ERIC-PCR fingerprinting performed on strains belonging to *Streptomyces. hydrogenans.* (B) BOX-PCR fingerprinting performed on strains belonging to *S. hydrogenans.*

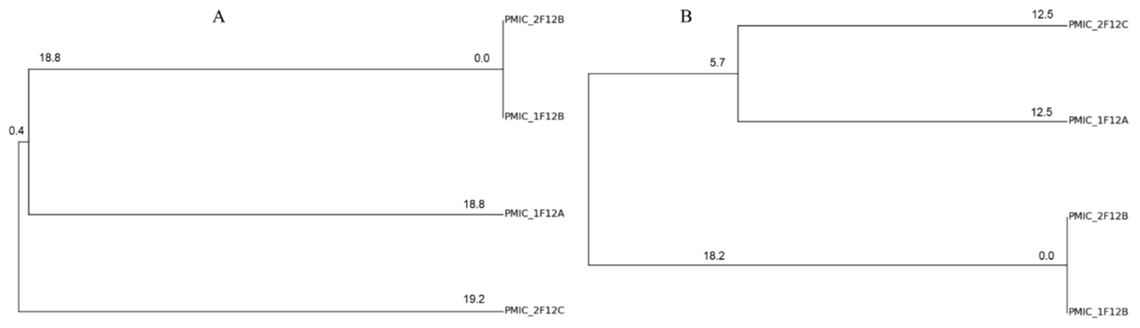

Figure S10 - (A) ERIC-PCR fingerprinting performed on strains belonging to *Streptomyces setonii.* (B) BOX-PCR fingerprinting performed on strains belonging to *S. setonii.*

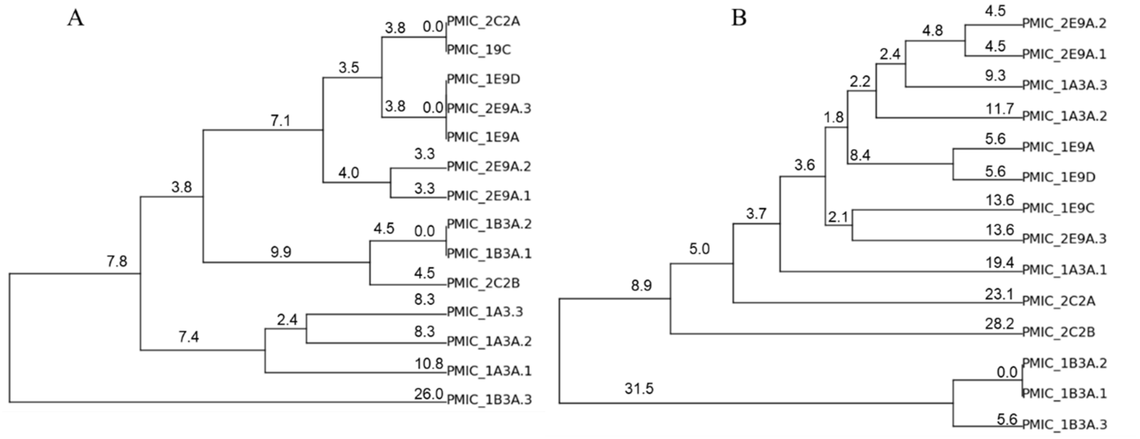

Figure S11 - (A) ERIC-PCR fingerprinting performed on strains belonging to *Streptomyces xiamenensis.* (B) BOX-PCR fingerprinting performed on strains belonging to *S. xiamenensis.*
